# Machine learning algorithm to predict mortality in critically ill patients with sepsis-associated acute kidney injury

**DOI:** 10.1038/s41598-023-32160-z

**Published:** 2023-03-30

**Authors:** Xunliang Li, Ruijuan Wu, Wenman Zhao, Rui Shi, Yuyu Zhu, Zhijuan Wang, Haifeng Pan, Deguang Wang

**Affiliations:** 1 Department of Nephrology, The Second Affiliated Hospital of Anhui Medical University, Anhui Medical University, Hefei, People’s Republic of China; 2 Institute of Kidney Disease, Inflammation and Immunity Mediated Diseases, The Second Affiliated Hospital of Anhui Medical University, Anhui Medical University, Hefei, People’s Republic of China; 3 Department of Epidemiology and Biostatistics, School of Public Health, Anhui Medical University, Hefei, People’s Republic of China; 4 Inflammation and Immune Mediated Diseases Laboratory of Anhui Province, Hefei, People’s Republic of China

**Keywords:** Medical research, Nephrology

## Abstract

This study aimed to establish and validate a machine learning (ML) model for predicting in-hospital mortality in patients with sepsis-associated acute kidney injury (SA-AKI). This study collected data on SA-AKI patients from 2008 to 2019 using the Medical Information Mart for Intensive Care IV. After employing Lasso regression for feature selection, six ML approaches were used to build the model. The optimal model was chosen based on precision and area under curve (AUC). In addition, the best model was interpreted using SHapley Additive exPlanations (SHAP) values and Local Interpretable Model-Agnostic Explanations (LIME) algorithms. There were 8129 sepsis patients eligible for participation; the median age was 68.7 (interquartile range: 57.2–79.6) years, and 57.9% (4708/8129) were male. After selection, 24 of the 44 clinical characteristics gathered after intensive care unit admission remained linked with prognosis and were utilized developing ML models. Among the six models developed, the eXtreme Gradient Boosting (XGBoost) model had the highest AUC, at 0.794. According to the SHAP values, the sequential organ failure assessment score, respiration, simplified acute physiology score II, and age were the four most influential variables in the XGBoost model. Individualized forecasts were clarified using the LIME algorithm. We built and verified ML models that excel in early mortality risk prediction in SA-AKI and the XGBoost model performed best.

## Introduction

Sepsis is a complicated medical condition caused by an infection that triggers a systemic inflammatory response^1^. It is the most common and dangerous cause of illness and death in critically ill people^2^. It is well known that sepsis frequently leads to acute kidney injury (AKI). AKI occurs in around 40% of people with severe sepsis, increasing the difficulty, cost, and likelihood of death during treatment^3–6^. Sepsis-associated acute kidney injury (SA-AKI) is a complicated condition, including multiple contributing factors associated with a worse prognosis, a longer length of hospital stay, and a more significant number of co-morbidities than in sepsis patients with no AKI^5–7^. It is crucial to accurately predict the prognosis for SA-AKI patients in the intensive care unit (ICU) due to their critical condition.

In critical care medicine, the prognosis of SA-AKI patients is a hot topic. Several scoring systems have been developed to predict outcomes in patients with SA-AKI; however, their performances have been disappointing due to low specificity and sensitivity. These scoring systems include the sequential organ failure assessment (SOFA) score, the simplified acute physiology score II (SAPS II), and the acute physiology and chronic health evaluation II^8,9^. Moreover, some multivariate prediction models for predicting the outcome of patients with SA-AKI have been developed. These models are based on standard statistical techniques like logistic regression and the Cox proportional risk model. Hu et al. used the Cox proportional risk model to construct a mortality prediction model for 2066 patients with SA-AKI, showing a preferable forecast performance^9^. However, the links between variables are intricate, including both linear and non-linear relationships; the Cox proportional risk model is, by default, calibrated to handle linear associations between dependent and independent variables, which may oversimplify more complex non-linear relationships. In addition, the Cox proportional risk model is susceptible to multicollinearity between variables, which might lower the model's performance. Consequently, it is crucial to investigate more effective and precise prediction techniques in the care of SA-AKI patients.

As statistical theory and computer technology have advanced, so has an interest in and acceptance of machine learning (ML) among medical professionals. Predictive models for various diseases have significantly benefited from cutting-edge ML approaches, outperforming their more conventional logistic and Cox regression-based counterparts^10,11^. The clinical applications of ML have ranged from diagnosis to prediction and have been utilized in various clinical domains^12–14^. ML methods have also been used to forecast the prognoses of critically ill patients, with results that are superior to those obtained using the more conventional methods of logistic regression and Cox regression analysis^15–18^. However, the advantage of ML algorithms in predicting mortality in SA-AKI patients has not yet been demonstrated. This research tried to create and verify ML models for early predicting in-hospital mortality in SA-AKI patients.

## Methods

### Database introduction

The Medical Information Mart for Intensive Care IV (MIMIC IV) database is an integrated, de-identified, and full clinical dataset that covers all patients who were hospitalized in the ICUs at Beth Israel Deaconess Medical Center in Boston, Massachusetts, between the years 2008 and 2019^19^. We acquired the certificate necessary to apply for database access after passing the exam to ensure the safety of human study participants (No. 35970146). Patient permission and an ethical approval statement were unnecessary because the experiment would not have affected clinical care, and all patient data had already been de-identified^20^. This research was carried out in conformity with the principles of the 2013 Helsinki Declaration.

### Study population

This study enrolled adults with sepsis who developed AKI within 48 h of ICU admission. Patients with sepsis were identified within 24 h of ICU admission using the Sepsis-3 criteria, which required the presence of both a probable infectious cause and a SOFA score ≥ 2^21^. AKI was diagnosed using the Kidney Disease: Improving Global Outcomes Clinical Practice Guideline’s (2012) suggested criteria of serum creatinine (Scr) and urine output^22^. The first available Scr following ICU admission was used as the baseline value if no Scr was available prior to admission^23^. Due to the importance of maintaining data independence, only the initial ICU hospitalization was included in the study if the patient had multiple admissions. Patients under the age of 18 or with ICU stays shorter than 48 h were excluded.

### Data collection

We gathered data on the patient’s demographic features, chronic disease history, vital signs, laboratory results, Treatments, illness severity scores, and outcomes.

Demographic features obtained for the research consisted of age, sex, and weight. Chronic disease history included chronic pulmonary disease, peptic ulcer disease, peripheral vascular disease, myocardial infarction, cerebrovascular disease, diabetes, acquired immune deficiency syndrome, renal disease, dementia, rheumatic disease, paraplegia, liver disease, cancer, and congestive heart failure. We gathered mean values for vital information, such as heart rate, mean arterial pressure, respiration rate, body temperature, and SpO_2_, in the first 24 h after ICU admission. The highest values for a variety of laboratory results were collected in the first 20 h following ICU admission. These tests included the Scr, serum glucose, serum chloride, serum calcium, hematocrit, hemoglobin, platelets, anion gap, white blood cell, international normalized ratio, bicarbonate, serum sodium, blood urea nitrogen, serum potassium, prothrombin time, and partial thromboplastin time. We also recorded the quantity of urine passed in the first 24 h after ICU admission. Treatments included using renal replacement therapy, vasopressors, and mechanical ventilation during the first 24 h after ICU admission. In the first 24 h following ICU admission, we analyzed the initial SOFA score and SAPS II to determine the severity of the patient’s conditions.

### Preprocessing of data

In this study, missing values for all variables were fewer than 20% (See Supplementary Table S1). When dealing with missing data, we employed the multiple imputation method implemented in the Python ‘miceforest’ package, widely acknowledged as a superior strategy for missing variables. We identified potential mortality-related variables using the least absolute shrinkage and selection operator (LASSO) analysis to reduce overfitting.

### Statistical analysis

The median and interquartile range (IQR) were used to describe the normal distribution of continuous variables, whereas numbers and percentages were used to describe categorical variables. If applicable, The Mann–Whitney or Student’s t-test. Comparing continuous variables between groups using the U test. Apply either the Pearson chi-squared or Fisher's exact test to evaluate the significance of group differences in categorical variables.

R version 4.2.1 and Python version 3.9.12 were used for all statistical analyses. A two-tailed *P* value below 0.05 was considered statistically significant.

### Machine learning

In this study, all participants were randomly divided into two sets a training set (consisting of 80% of patients) and a validation set (consisting of 20% of patients) (See Supplementary Table S2). The dimension of the features was reduced using the LASSO technique. Six different ML methods—logistic regression, support vector machine (SVM), k-nearest neighbor (KNN), decision tree, random forest (RF), and extreme gradient boosting (XGBoost)—were used to create and test models for predicting risks of in-hospital mortality. The ML model prediction ability was measured using accuracy, area under curve (AUC), sensitivity, specificity, and average precision. After considering accuracy and AUC, we settled on our final candidate model. To compare the predictive power between the models, we performed decision curve analysis (DCA) and plotted calibration curves. Using SHapley Additive exPlanations (SHAP) values, we characterized the crucial characteristics that affect mortality risk in the best ML model to study further each characteristic’s significance to the optimal model’s output. At last, the Local Interpretable Model-Agnostic Explanations (LIME) technique is used to fit the model's expected behavior. Finally, a sensitivity analysis of the results was performed.

### Ethics approval and consent to participate

MIMIC IV was set up with the approval of the Institutional Review Board at the Massachusetts Institute of Technology. All participant data were anonymized to safeguard their privacy. Due to the use of anonymized health records, ethical approval and informed consent were not required. This study adheres to the ethical criteria outlined in the Helsinki Declaration of 1964.

## Results

### Participants

**Figure 1:**
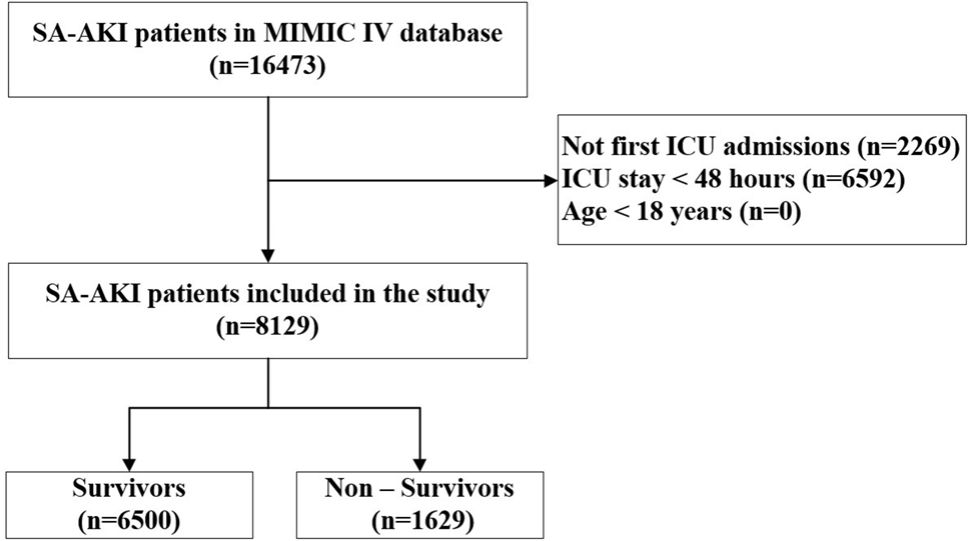
The flowchart of patient selection. *MIMIC IV* Medical information mort for intensive care IV, *ICU* Intensive care unit, *SA-AKI* Sepsis-associated acute kidney injury.

The total number of people with SA-AKI who were considered for inclusion was 16,473. However, 2269 were ruled out because they had more than one ICU hospitalization, and 6592 excluded for less than 48 h in ICU. In the end, 8129 patients qualified for the study (Fig. 1). The prevalence of death in hospitals was 20% (1629/8129). The median age of these patients was 68.7 (IQR: 57.2–79.6) years, and 57.9% (4708/8129) were male. The top three comorbidities were congestive heart failure (2831/8129, 34.8%), diabetes (2566/8129, 31.6%), and chronic pulmonary illness (2358/8129, 29.0%). Table 1 provides a summary of the base characteristics of the dataset.

**Table 1:** Demographic and clinical characteristics at baseline.

Variables	Total (n = 8129)	Survivors (n = 6500)	Non-survivor (n = 1629)	*P* value
Demographic features				
Age (years)	68.7 [57.2, 79.6]	68.1 [56.8, 79.0]	71.3 [59.3, 81.8]	< 0.001
Sex, male, n (%)	4708 (57.9)	3806 (58.6)	902 (55.4)	0.022
Weight (kg)	81.8 [68.7, 98.0]	82.6 [69.5, 98.8]	79.0 [65.5, 95.0]	< 0.001
Chronic disease history, n (%)				
Chronic pulmonary disease	2358 (29.0)	1885 (29.0)	473 (29.0)	1
Peptic ulcer disease	261 (3.2)	200 (3.1)	61 (3.7)	0.198
Peripheral vascular disease	1143 (14.1)	915 (14.1)	228 (14.0)	0.965
Myocardial infarction	1643 (20.2)	1302 (20.0)	341 (20.9)	0.437
Cerebrovascular disease	1250 (15.4)	915 (14.1)	335 (20.6)	< 0.001
Diabetes	2566 (31.6)	2088 (32.1)	478 (29.3)	0.033
Aids	37 (0.5)	29 (0.4)	8 (0.5)	0.972
Renal disease	1938 (23.8)	1508 (23.2)	430 (26.4)	0.007
Dementia	321 (3.9)	242 (3.7)	79 (4.8)	0.044
Rheumatic disease	305 (3.8)	229 (3.5)	76 (4.7)	0.036
Paraplegia	376 (4.6)	277 (4.3)	99 (6.1)	0.002
Liver disease	1384 (17.0)	921 (14.2)	463 (28.4)	< 0.001
Cancer	1108 (13.6)	774 (11.9)	334 (20.5)	< 0.001
Congestive heart failure	2831 (34.8)	2236 (34.4)	595 (36.5)	0.114
Vital signs				
Heart rate (beats/minute)	86.1 [76.2, 98.5]	85.3 [76.2, 97.3]	89.6 [76.6, 102.8]	< 0.001
MAP (mmHg)	74.8 [69.6, 81.3]	75.0 [69.9, 81.4]	74.1 [68.2, 80.9]	< 0.001
Respiratory rate (beats/minute)	19.3 [16.9, 22.4]	19.0 [16.7, 22.0]	20.9 [18.1, 24.0]	< 0.001
Body temperature (°C)	36.9 [36.6, 37.3]	36.9 [36.6, 37.3]	36.8 [36.4, 37.2]	< 0.001
SpO_2_ (%)	97.4 [95.9, 98.7]	97.5 [96.0, 98.7]	97.2 [95.5, 98.7]	< 0.001
Laboratory results				
Scr (mg/dL)	1.3 [0.9, 2.1]	1.2 [0.9, 2.0]	1.7 [1.1, 2.6]	< 0.001
Serum glucose (mg/dL)	155 [124, 209]	151 [122, 200]	173 [132, 238]	< 0.001
Serum chloride (mEq/L)	107 [103, 111]	107 [103, 111]	106 [101, 111]	< 0.001
Serum calcium (mg/dL)	8.5 [8.0, 9.0]	8.5 [8.0, 8.9]	8.5 [8.0, 9.1]	0.002
Hematocrit (%)	34.8 [30.7, 39.7]	35.0 [31.0, 39.6]	34.4 [29.7, 40.1]	0.004
Hemoglobin (g/dL)	11.4 [10.0, 13.1]	11.5 [10.1, 13.1]	11.2 [9.6, 13.0]	< 0.001
Platelets (K/uL)	209 [151, 282]	210 [155, 281]	202 [130, 288]	< 0.001
Anion gap (mEq/L)	17.0 [14.0, 20.0]	16.0 [13.0, 19.0]	19.0 [15.0, 22.0]	< 0.001
WBC (K/uL)	14.6 [10.6, 19.8]	14.4 [10.6, 19.4]	15.7 [11.1, 21.6]	< 0.001
INR	1.4 [1.2, 1.7]	1.3 [1.2, 1.6]	1.5 [1.2, 2.2]	< 0.001
Bicarbonate (mmol/L)	24.0 [21.0, 27.0]	24.0 [22.0, 27.0]	23.0 [20.0, 26.0]	< 0.001
Serum sodium (mEq/L)	140 [137, 143]	140 [138, 143]	140 [137, 144]	0.056
BUN (mg/dL)	26.0 [17.0, 42.0]	24.0 [16.0, 39.0]	33.0 [22.0, 53.0]	< 0.001
Serum potassium (mEq/L)	4.6 [4.2, 5.1]	4.5 [4.2, 5.1]	4.6 [4.2, 5.3]	< 0.001
PT (s)	15.2 [13.2, 18.7]	14.9 [13.1, 17.9]	16.5 [13.6, 23.5]	< 0.001
PTT (s)	34.2 [29.0, 48.9]	33.5 [28.7, 46.3]	38.5 [30.3, 58.2]	< 0.001
Urine output (mL)	1295 [765, 2000]	1385 [855, 2090]	915 [445, 1598]	< 0.001
Treatments, n (%)				
RRT	592 (7.3)	408 (6.3)	184 (11.3)	< 0.001
Vasopressors use	785 (9.7)	498 (7.7)	287 (17.6)	< 0.001
Mechanical ventilation	7485 (92.1)	5980 (92.0)	1505 (92.4)	0.64
Severity scores of illness				
SOFA score	7 [5, 10]	7 [4, 10]	10 [7, 13]	< 0.001
SAPS II	42 [34, 52]	40 [32, 49]	51 [41, 61]	< 0.001

*AIDS* Acquired immune deficiency syndrome, *MAP* Mean arterial pressure, *SpO*_*2*_ Oxygen saturation, *WBC* White blood cell, *BUN* Blood urea nitrogen, *INR* International normalized ratio, *PT* Prothrombin time, *PTT* Partial thromboplastin time, *RRT* Renal replacement therapy, *Scr* Serum creatinine, *SOFA* Sequential organ failure assessment, *SAPS II* Simplified acute physiology score II.

### Predictor selection

Multiple imputations were employed to fill in missing values for each variable. On the first day after being admitted to the ICU, 44 variables were gathered and analyzed using LASSO regression. Twenty-four variables were found to be statistically significant predictors of mortality after feature selection with LASSO analysis (See Supplementary Fig. S1), and these are listed in detail in Supplementary Table S3.

### Model development and validation

**Figure 2:**
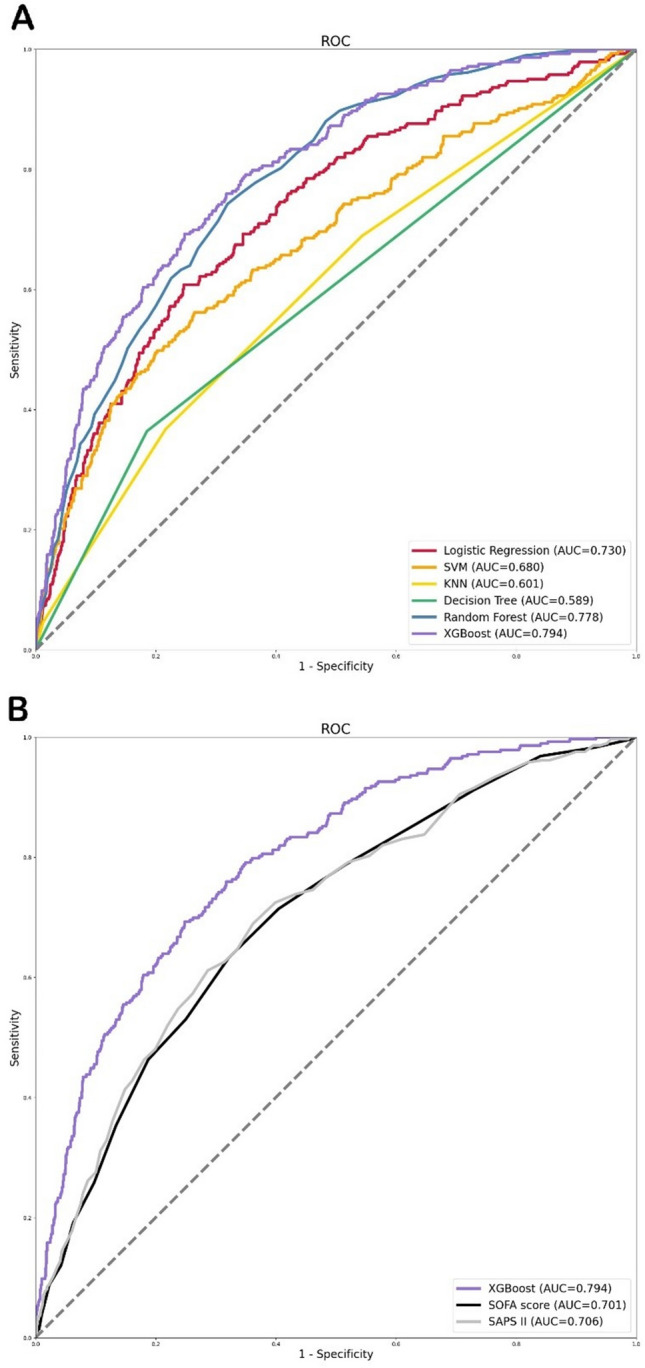
ROC curves for the ML models and the traditional severity of illness scores to predict in-hospital mortality. (**A**) ROC curves for the six ML models used to predict in-hospital mortality; (**B**) ROC curves for the traditional severity of disease scores used to predict in-hospital mortality. *ROC* Receiver operating characteristic, *SVM* Support vector machine, *KNN* k-nearest neighbors, *AUC* Area under the curve, *SOFA* Sequential organ failure assessment, *SAPS II* Simplified acute physiology score II.

The total number of patients was 8129, and they were divided randomly between a training group of 6503 (80%) and a validation cohort of 1626 (20%). There were no significant differences in the baseline features between the training and validation sets. Using LASSO regression’s selected 24 variables, we built six ML models: logistic regression, SVM, KNN, decision tree, RF, and XGBoost. The XGBoost model achieved the highest AUC (0.794) in the validation cohort, outperforming logistic regression (0.730), SVM (0.680), KNN (0.601), decision tree (0.585), and RF (0.778) (Fig. 2A). In order to dig even deeper into the performance of the six models, we also measured their accuracy, sensitivity, specificity, and average precision, and the outcomes are tabulated in Table 2. Other clinical disease severity ratings [SOFA score (AUC: 0.701); SAPS II (AUC: 0.706)] did not perform as well as the XGBoost model (Fig. 2B). The DCA curves and calibration curves show that the XGBoost model performs best among the six models (See Supplementary Figs. S2 and S3).

**Table 2:** Performance comparison of the machine learning models in the validation set.

Models	Accuracy	AUC (95% CI)	Sensitivity	Specificity	Average precision
Logistic regression	0.822	0.730 (0.694–0.765)	0.608	0.754	0.572
Support vector machine	0.826	0.680 (0.643–0.717)	0.562	0.736	0.556
k-Nearest neighbor	0.793	0.601 (0.563–0.638)	0.367	0.783	0.429
Decision tree	0.737	0.585 (0.547–0.623)	0.378	0.812	0.425
Random forest	0.825	0.778 (0.745–0.812)	0.739	0.674	0.622
XGBoost	0.832	0.794 (0.762–0.827)	0.793	0.752	0.660

*AUC* Area under curve, *CI* Confidence interval, *XGBoost* Extreme gradient boosting.

### Model explainability

**Figure 3:**
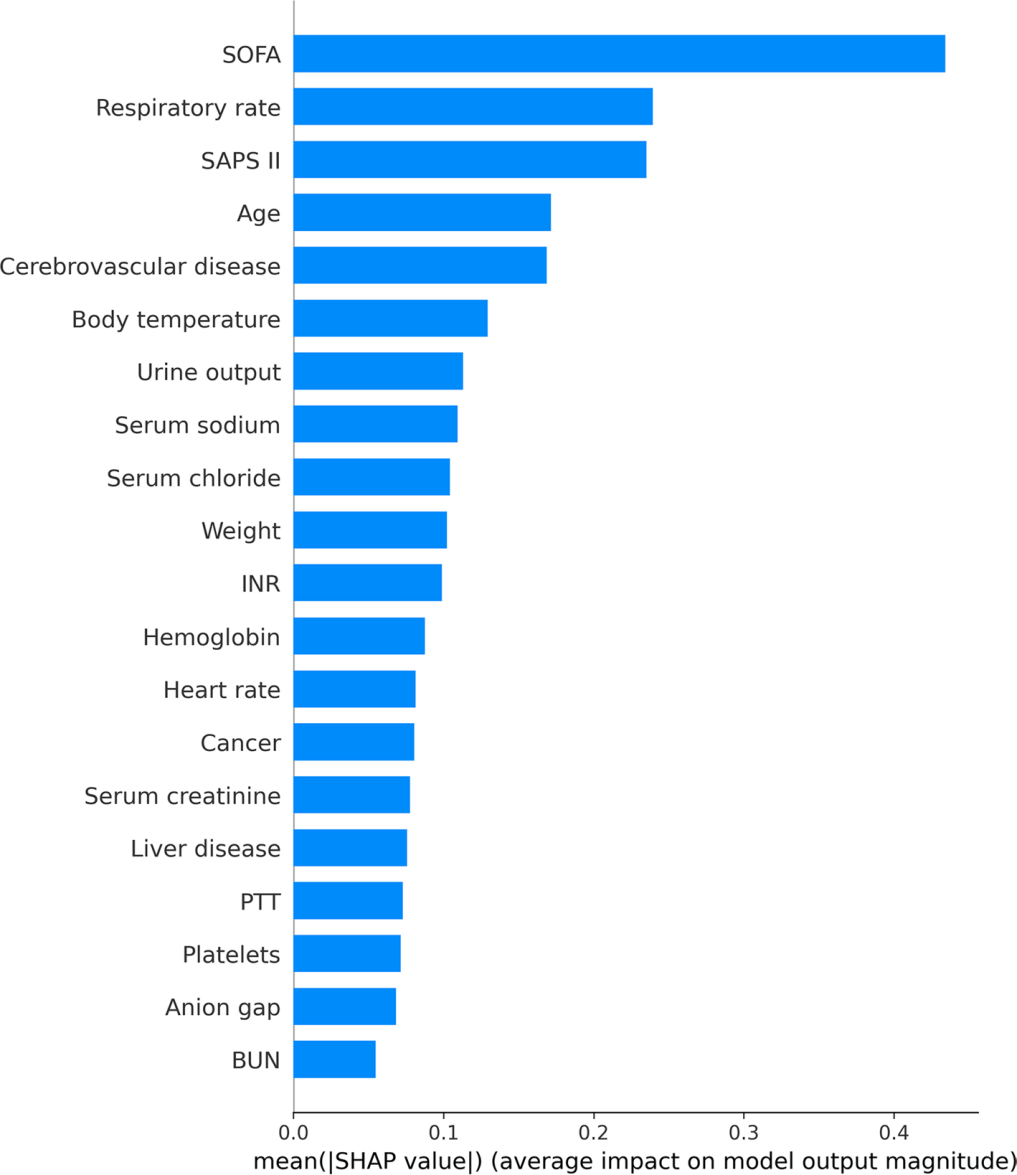
The top 20 important features derived from the XGBoost model. SHAP indicates the importance ranking of features. The significance of each covariate in the construction of the final predictive model is represented by the matrix plot. *SHAP* SHapley additive explanation, *SOFA* Sequential organ failure assessment, *SAPS II* Simplified acute physiology score II, *INR* International normalized ratio, *PTT* Partial thromboplastin time, *BUN* Blood urea nitrogen.

**Figure 4:**
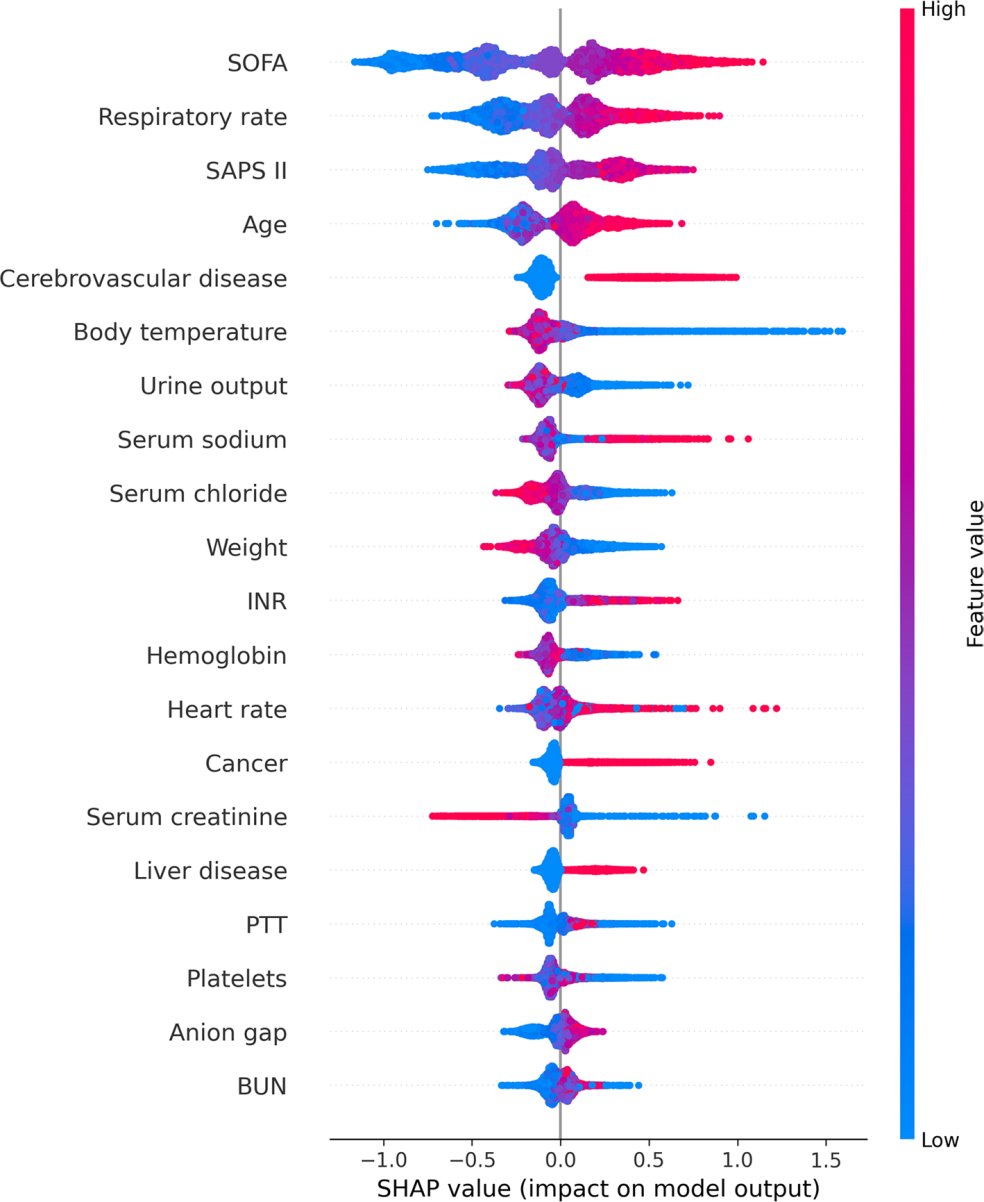
SHAP summary plot of the top 20 features of the XGBoost model. The greater the SHAP value of a characteristic, the greater the likelihood of death development. The abscissa represents the SHAP value, and each line represents a feature. Red dots indicate greater feature values, whereas blue dots indicate lower feature values. *SHAP* SHapley additive explanation, *SOFA* Sequential organ failure assessment, *SAPS* II Simplified acute physiology score II, *INR* International normalized ratio, *PTT* Partial thromboplastin time, *BUN* Blood urea nitrogen.

**Figure 5:**
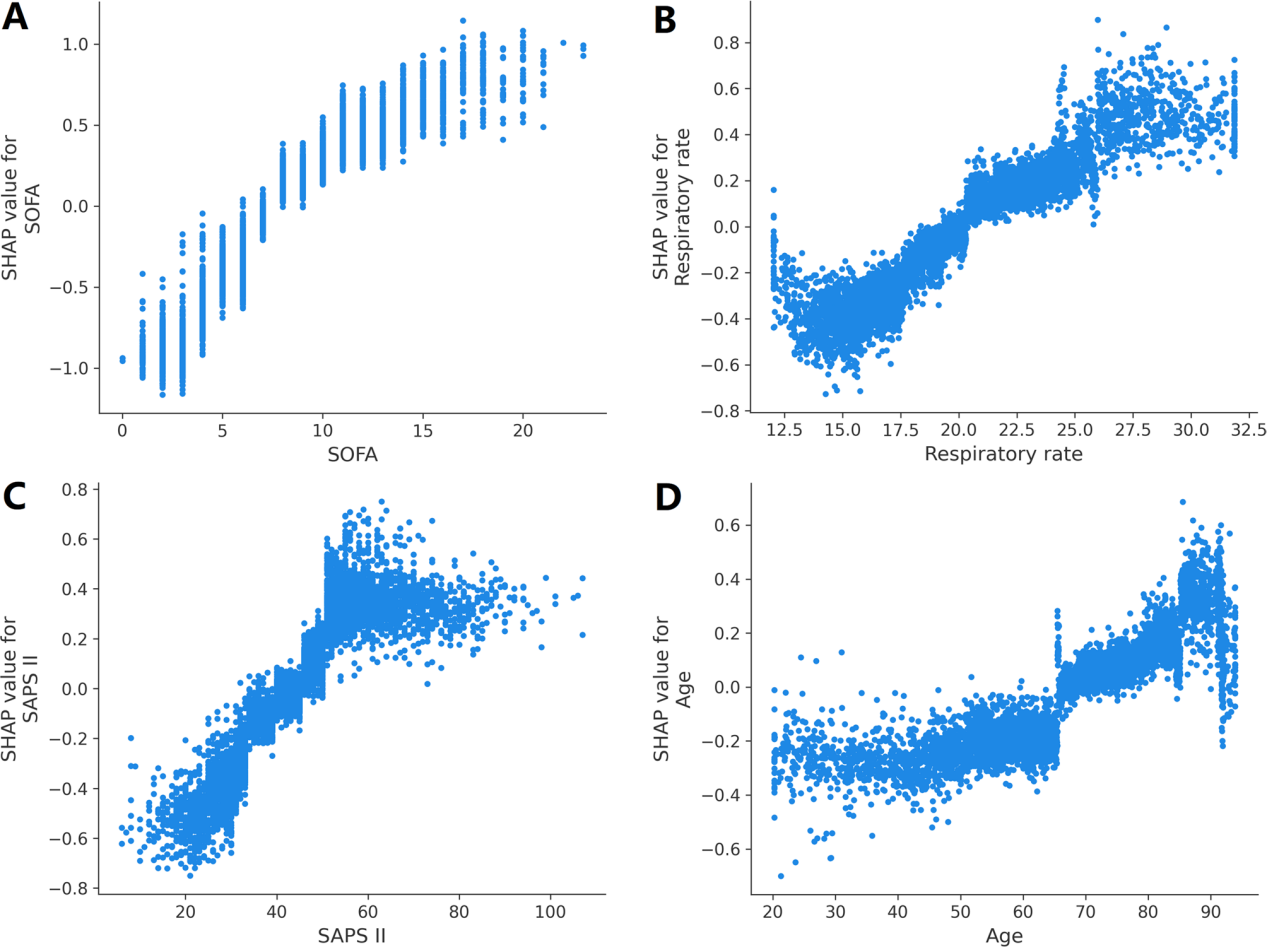
SHAP dependence plot of the XGBoost model. (**A**) SOFA score; (**B**) Respiratory rate; (**C**) SAPS II; (**D**) Age. Certain SHAP levels surpass zero indicates an elevated risk of death. *SHAP* SHapley additive explanation, *SOFA* Sequential organ failure assessment, *SAPS II* Simplified acute physiology score II.

With SHAP values, we hoped to provide more insight into how the XGBoost model predicts deaths. For the XGBoost model, the SHAP summary graphic reveals that the SOFA score, respiratory rate, SAPS II, and age are the four most significant parameters (Fig. 3). In addition, we used SHAP dependence analysis to illustrate the impact of a single input variable had on the XGBoost prediction model’s final results (Fig. 4). Figure 5 displays the findings of a more in-depth analysis of the four most influential clinical features on the XGBoost prediction model's output.

**Figure 6:**
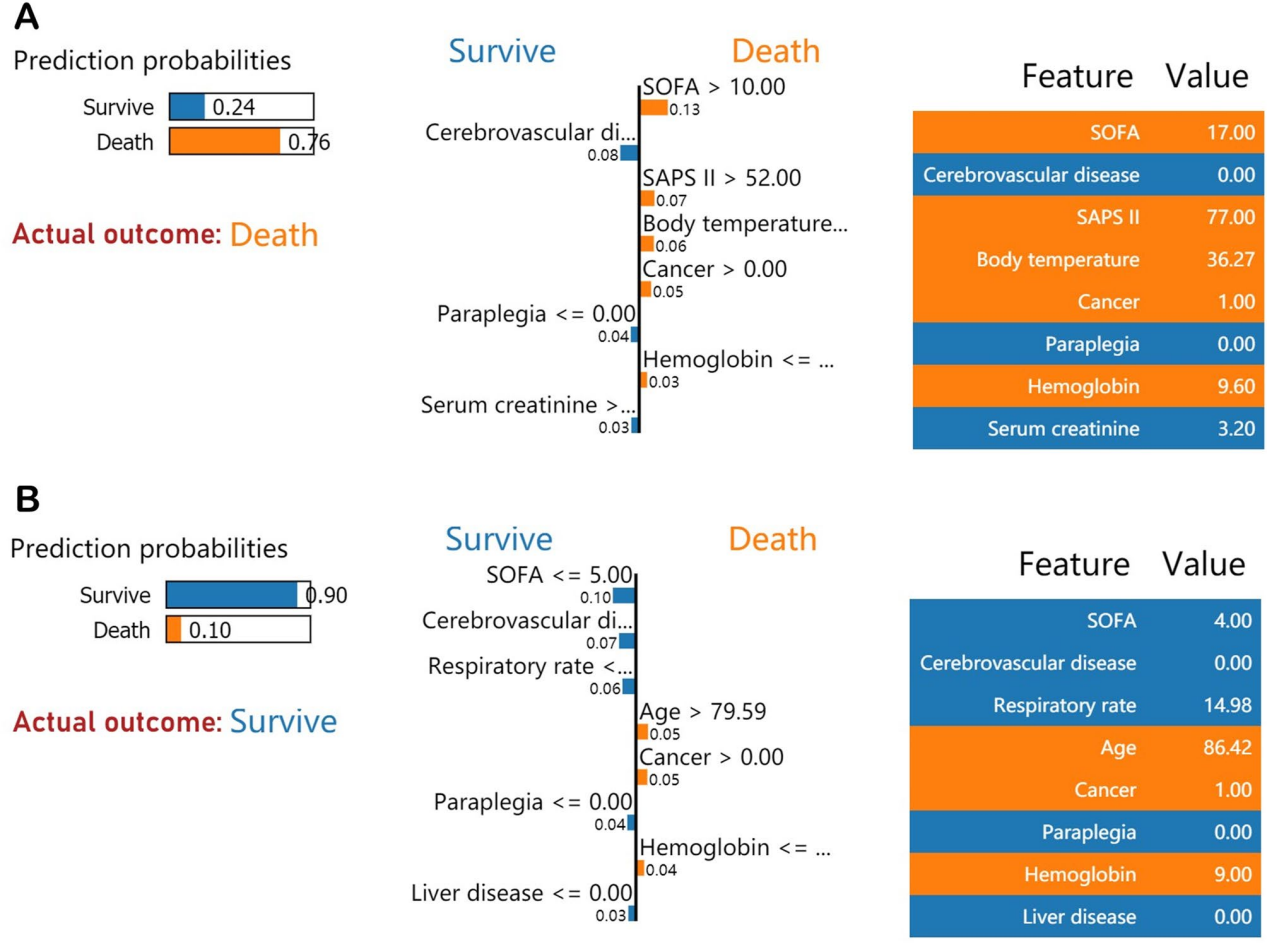
LIME algorithm for explaining individual’s prediction results. Screenshot of the death prognosis for SA-AKI patients. (**A**) Utilizing the LIME method, show a death case. (**B**) Present a case of survival using the LIME method. The left portion of the picture depicts expected LIME findings. The center section lists, from highest to lowest, the eight variables that had the greatest impact on survival or death. The length of the bar for each feature reflects the weight of that feature in the prediction. A longer bar represents a characteristic that contributes more to survival or mortality. The right panel displays the crucial values of these eight factors at which they had the greatest influence on survival or death. *SA-AKI* Sepsis-associated acute kidney injury, *SOFA* Sequential organ failure assessment, *SAPS II* Simplified acute physiology score II, *SpO*_*2*_ Oxygen saturation, *PTT* Partial thromboplastin time, *WBC* White blood cell.

We then took two random samples from the validation set and ran them through the LIME algorithm to shed light on the individual mortality forecast. Figure 6A depicts the case of death reported by the LIME algorithm. 76% was the expected probability of death according to the XGBoost model. The XGBoost model found a SOFA score of 17, a SAPS II of 77, a temperature of 36.27 °C, a history of malignancy, and a hemoglobin level of 9.6 g/dL were all associated with an elevated risk of mortality. Scr levels below 3.2 mg/dL and the absence of a prior history of cerebrovascular illness or paraplegia were found to reduce mortality risk. Both the XGBoost model and the actual outcome for this patient were death. Similarly, Fig. 6B illustrates a survival example utilizing the LIME technique. 10% was the expected probability of death according to the XGBoost model. The patient's age of 86.42 years, the history of cerebrovascular illness, and the hemoglobin level of 9 g/dL increase the risk of mortality, whereas a SOFA score of 4, the absence of a history of cerebrovascular disease, a respiratory rate of 14.98 beats per minute, the absence of a history of paraplegia, and the absence of a history of liver disease decrease the risk of death. Both the actual and expected outcomes confirmed the XGBoost model's prediction of the patient’s survival.

### Sensitivity analyses

For patients without renal disease (N = 1232), the XGBoost model remained robust in predicting mortality in these patients (AUC: 0.808). Detailed results are shown in Supplementary Fig. S4.

## Discussion

This study developed and verified six ML methods for estimating the risk of in-hospital death among patients with SA-AKI. In predicting mortality in patients with SA-AKI, the XGBoost models outperformed other ML models (such as logistic regression, SVM, KNN, decision tree, and RF models) and conventional risk scores (such as the SOFA score and SAPS II). After doing a feature importance analysis, we found that the SOFA score, respiratory rate, SAPS II, and age were the top 4 features of the XGBoost model in terms of their ability to predict mortality. Also, we have documented how these factors influenced the XGBoost model. Finally, we use the LIME algorithm for personalized forecasting.

SA-AKI is frequent in critically ill patients and is characterized by rapid clinical deterioration and a considerably greater mortality rate than patients without AKI or those with other variables producing AKI^24^. Clinicians require accurate prediction models to evaluate the risk of dying and make appropriate therapy choices for severely ill patients with SA-AKI. For outcome prediction in intensive care settings, generic metrics such as the SOFA score and SAPS II are widely applied. The SOFA and SAPS II scoring systems have a number of shortcomings compared to ML models, including unsatisfactory predictive performance, poor specificity and sensitivity, a wide range of variability, and a laborious procedure^10^. According to the results of this research, the conventional severity scoring methods performed poorly compared to the ML model. This could be due to the two reasons listed below. First, the risk of bad outcomes in critically sick patients was assessed using the SOFA and SAPS II scoring systems, which relied heavily on the practitioner’s prior expertise^25^. Second, unlike multivariate models, these scoring systems cannot analyze a large number of potentially valuable variables, reducing their predictive power^26^.

Consistent with previous studies, our results show that the XGBoost model outperforms the other ML models in predicting death in SA-AKI patients. Liu et al. found that the XGBoost model predicted mortality in AKI patients better than logistic regression, SVM, and RF^27^. Zhu et al. evaluated the prediction of hospital mortality for patients on mechanical ventilation and discovered that the XGBoost model performed better than the RF, logistic regression, decision tree, and KNN models^28^. It is possible that multiple factors contributed to the boost in prediction abilities seen in XGBoost models. Firstly, the XGBoost method, derived from the gradient tree boosting framework, is highly skilled at fitting high-order interactions, discontinuities, and non-linearities. Second, the XGBoost method is resistant to outliers in the predictor variables and multicollinearity among those variables.

To enhance the interpretability of the model, we employ the SHAP value to explain and show the most influential elements of the prediction results. This study's analysis of the XGBoost model’s summary of feature importance found that the SOFA score was the most important predictor of mortality in patients with SA-AKI. The SOFA score quantifies organ impairment by measuring the burden of organ malfunction. The SOFA score was found to have a significant correlation with clinical outcomes, with a high SOFA score typically suggesting a critical condition and poor prognosis. Despite this, none of the prior models predicted the probability of mortality for SA-AKI patients using this crucial factor^9,28^. The SOFA score was the most heavily weighted variable in the XGBoost model, showing its significance in predicting the mortality of patients with SA-AKI in the present study. Additionally, we found that the rate of breathing is significantly correlated with the risk of dying from SA-AKI. Some research has demonstrated a correlation between breathing rate and inferior outcomes until the present day^29^. The SAPS II was an additional influential predictor of SA-AKI outcomes. SAPS II is a measure of disease severity, and greater SAPS II scores are linked to higher in-hospital death in critically sick patients^30^. Our results also showed that age was a significant risk factor for death among critically ill patients identified with SA-AKI. In the absence of co-morbidities and advanced age, the mortality rate from sepsis is less than 5%, according to a study conducted in Australia and New Zealand^31^.

However, this study also had some shortcomings. First, our study may be subject to selection bias because of its retrospective and observational design. Second, the current study was limited in its ability to conclude cause and effect because it was a retrospective modeling study conducted at a single location utilizing the MIMIC IV database. That being said, more prospective randomized clinical trials are needed to verify our model's efficacy. Third, we estimated specific missing data using the filling method, which may result in divergence from the genuine value. Finally, this study only validated the models internally; therefore, multicenter external validation is still needed to assess the models’ predictive ability. Finally, this study only validated the models internally; therefore, multicenter external validation is still required to assess the predictive potential of the models.

## Conclusion

We built and verified ML models that excel in early mortality risk prediction in SA-AKI. The XGBoost model is the most effective of all the algorithms. Further SHAP values and LIME method indicated that SOFA score, respiratory rate, SAPS II, and age were played as the marked contributors for the prediction of death in SA-AKI patients. These findings would be helpful for clinical prediction. However, multi-center studies are still necessary to ensure that if this ML model are broadly applicable and generalizable to various settings and associated with improved clinical decision-making and outcomes.

## Supplementary Information

Supplementary Information.

## Data Availability

The datasets presented in the current study are available in the MIMIC IV database (https://physionet.org/content/mimiciv/1.0/).
