# Supplementary material for: Machine learning algorithm to predict mortality in critically ill patients with sepsis-associated acute kidney injury
Source: Sci Rep. 2023 Mar 30;13:5223. doi: 10.1038/s41598-023-32160-z (PMC10063657; doi:10.1038/s41598-023-32160-z)
Supplement: Supplementary file 1 — Supplementary Information. [file 41598_2023_32160_MOESM1_ESM.docx]

**Machine learning algorithm to predict mortality in critically ill patients with sepsis-associated acute kidney injury**

Xunliang Li^1,2,*^, Ruijuan Wu^1,2,*^, Wenman Zhao^1,2^, Rui Shi^1,2^, Yuyu Zhu^1,2^, Zhijuan Wang^1,2^, Haifeng Pan^2,3,4^ and Deguang Wang^1,2^

^1^Department of Nephrology, The Second Hospital of Anhui Medical University, Anhui Medical University, Hefei, People’s Republic of China

^2^Institute of kidney disease, inflammation & immunity mediated Diseases, The Second Hospital of Anhui Medical University, Anhui Medical University, Hefei, People’s Republic of China

^3^Department of Epidemiology and Biostatistics, School of Public Health, Anhui Medical University, Hefei, People’s Republic of China

^4^Inflammation and Immune Mediated Diseases Laboratory of Anhui Province, Hefei, People’s Republic of China

**Table S1. Missing number (%) for variables**

| **Variables** | Missing number (%) |
| --- | --- |
| **Demographic features** |  |
| Age | 0(0) |
| Sex, male | 0(0) |
| Weight | 174(2.1%) |
|  |  |
| **Chronic disease history** | 0(0) |
| Chronic pulmonary disease | 0(0) |
| Peptic ulcer disease | 0(0) |
| Peripheral vascular disease | 0(0) |
| Myocardial infarction | 0(0) |
| Cerebrovascular disease | 0(0) |
| Diabetes | 0(0) |
| Aids | 0(0) |
| Renal disease | 0(0) |
| Dementia | 0(0) |
| Rheumatic disease | 0(0) |
| Paraplegia | 0(0) |
| Liver disease | 0(0) |
| Cancer | 0(0) |
| Congestive heart failure | 0(0) |
|  |  |
| **Vital signs** |  |
| Heart rate | 1(0%) |
| MAP | 1(0%) |
| Respiratory rate | 3(0%) |
| Body temperature | 529(6.5%) |
| SpO_2_ | 1(0%) |
|  |  |
| **Laboratory results** |  |
| Scr | 7(0%) |
| Serum glucose | 39(0%) |
| Serum chloride | 7(0%) |
| Serum calcium, | 613(7.5%) |
| Hematocrit | 7(0%) |
| Hemoglobin | 8(0%) |
| Platelets | 9(0%) |
| Anion gap | 21(0%) |
| WBC | 8(0%) |
| INR | 274(3.4%) |
| Bicarbonate | 8(0%) |
| Serum sodium | 10(0%) |
| BUN | 9(0%) |
| Serum potassium | 14(0%) |
| PT | 274(3.4%) |
| PTT | 294(3.6%) |
|  |  |
| **Urine output** | 146(1.8%) |
|  |  |
| **Treatments** |  |
| RRT | 0(0) |
| Vasopressors use | 0(0) |
| Mechanical ventilation | 0(0) |
|  |  |
| **Severity scores of illness** |  |
| SOFA | 0(0) |
| SAPS II | 0(0) |

Abbreviations: Aids: acquired immune deficiency syndrome, MAP: mean arterial pressure, SpO_2_: oxygen saturation, WBC: white blood cell, BUN: blood urea nitrogen, INR: international normalized ratio, PT: prothrombin time, PTT: partial thromboplastin time, RRT: renal replacement therapy, SOFA: sequential organ failure assessment, Scr: serum creatinine, SAPS II: Simplified Acute Physiology Score II.

**Table S2. Comparison of baseline characteristics between the training and test sets**

| **Variables** | **Total**  **(n =8129)** | **Training set**  **(n =6503)** | **Test set**  **(n =1626)** | **P value** |
| --- | --- | --- | --- | --- |
| **Demographic features** |  |  |  |  |
| Age (years) | 68.7 [57.2, 79.6] | 68.7 [57.3, 79.6] | 68.7 [57.1, 79.6] | 0.534 |
| Sex, male, n (%) | 4708 (57.9) | 3760 (57.8) | 948 (58.3) | 0.745 |
| Weight (kg) | 81.8 [68.7, 98.0] | 81.6 [68.5, 98.0] | 82.1 [69.3, 97.6] | 0.637 |
|  |  |  |  |  |
| **Chronic disease history, n (%)** |  |  |  |  |
| Chronic pulmonary disease | 2358 (29.0) | 1877 (28.9) | 481 (29.6) | 0.589 |
| Peptic ulcer disease | 261 (3.2) | 206 (3.2) | 55 (3.4) | 0.718 |
| Peripheral vascular disease | 1143 (14.1) | 915 (14.1) | 228 (14.0) | 0.992 |
| Myocardial infarction | 1643 (20.2) | 1312 (20.2) | 331 (20.4) | 0.898 |
| Cerebrovascular disease | 1250 (15.4) | 999 (15.4) | 251 (15.4) | 0.971 |
| Diabetes | 2566 (31.6) | 2061 (31.7) | 505 (31.1) | 0.643 |
| Aids | 37 (0.5) | 29 (0.4) | 8 (0.5) | 0.967 |
| Renal disease | 1938 (23.8) | 1544 (23.7) | 394 (24.2) | 0.703 |
| Dementia | 321 (3.9) | 255 (3.9) | 66 (4.1) | 0.854 |
| Rheumatic disease | 305 (3.8) | 242 (3.7) | 63 (3.9) | 0.828 |
| Paraplegia | 376 (4.6) | 300 (4.6) | 76 (4.7) | 0.969 |
| Liver disease | 1384 (17.0) | 1097 (16.9) | 287 (17.7) | 0.476 |
| Cancer | 1108 (13.6) | 886 (13.6) | 222 (13.7) | 1 |
| Congestive heart failure | 2831 (34.8) | 2239 (34.4) | 592 (36.4) | 0.142 |
|  |  |  |  |  |
| **Vital signs** |  |  |  |  |
| Heart rate (beats/minute) | 86.1 [76.2, 98.5] | 86.2 [76.4, 98.6] | 85.4 [75.6, 98.1] | 0.424 |
| MAP (mmHg) | 74.8 [69.6, 81.3] | 74.8 [69.5, 81.3] | 74.9 [69.7, 81.4] | 0.736 |
| Respiratory rate (beats/minute) | 19.3 [16.9, 22.4] | 19.3 [17.0, 22.5] | 19.3 [16.8, 22.4] | 0.32 |
| Body temperature (°C) | 36.9 [36.6, 37.3] | 36.9 [36.6, 37.2] | 36.9 [36.6, 37.3] | 0.873 |
| SpO_2_ (%) | 97.4 [95.9, 98.7] | 97.4 [95.9, 98.7] | 97.4 [95.9, 98.7] | 0.666 |
|  |  |  |  |  |
| **Laboratory results** |  |  |  |  |
| Scr (mg/dL) | 1.3 [0.9, 2.1] | 1.3 [0.9, 2.1] | 1.2 [0.9, 2.1] | 0.423 |
| Serum glucose (mg/dL) | 155 [124, 209] | 155 [124, 208] | 155 [124, 212] | 0.637 |
| Serum chloride (mEq/l) | 107 [103, 111] | 107 [103, 111] | 107 [103, 111] | 0.212 |
| Serum calcium (mg/dL) | 8.5 [8.0, 9.0] | 8.5 [8.0, 9.0] | 8.5 [8.0, 9.0] | 0.118 |
| Hematocrit (%) | 34.8 [30.7, 39.7] | 34.8 [30.7, 39.7] | 35.1 [30.9, 39.7] | 0.401 |
| Hemoglobin (g/dL) | 11.4 [10.0, 13.1] | 11.4 [10.0, 13.1] | 11.5 [10.1, 13.1] | 0.353 |
| Platelets (K/uL) | 209 [151, 282] | 208 [151, 281] | 213 [151, 286] | 0.282 |
| Anion gap (mEq/L) | 17.0 [14.0, 20.0] | 17.0 [14.0, 20.0] | 16.5 [14.0, 20.0] | 0.603 |
| WBC (K/uL) | 14.6 [10.6, 19.8] | 14.6 [10.6, 19.7] | 14.7 [10.9, 20.2] | 0.081 |
| INR | 1.4 [1.2, 1.7] | 1.40 [1.2, 1.7] | 1.4 [1.2, 1.7] | 0.171 |
| Bicarbonate (mmol/L) | 24.0 [21.0, 27.0] | 24.0 [21.0, 27.0] | 24.0 [22.0, 26.0] | 0.782 |
| Serum sodium (mEq/L) | 140 [137, 143] | 140 [137, 143] | 140 [138, 143] | 0.093 |
| BUN (mg/dL) | 26.0 [17.0, 42.0] | 26.0 [17.0, 42.0] | 25.0 [17.0, 42.0] | 0.282 |
| Serum potassium (mEq/L) | 4.6 [4.2, 5.1] | 4.60 [4.2, 5.1] | 4.60 [4.2, 5.1] | 0.965 |
| PT (s) | 15.2 [13.2, 18.7] | 15.2 [13.3, 18.8] | 15.0 [13.2, 18.6] | 0.189 |
| PTT (s) | 34.2 [29.0, 48.9] | 34.3 [29.0, 48.8] | 34.1 [28.9, 49.3] | 0.655 |
|  |  |  |  |  |
| **Urine output (mL)** | 1295 [765, 2000] | 1280 [760, 1985] | 1350 [794, 2050] | 0.029 |
|  |  |  |  |  |
| **Treatments, n (%)** |  |  |  |  |
| RRT | 592 (7.3) | 475 (7.3) | 117 (7.2) | 0.922 |
| Vasopressors use | 785 (9.7) | 647 (9.9) | 138 (8.5) | 0.082 |
| Mechanical ventilation | 7485 (92.1) | 5978 (91.9) | 1507 (92.7) | 0.339 |
|  |  |  |  |  |
| **Severity scores of illness** |  |  |  |  |
| SOFA | 7 [5, 10] | 7 [5, 10] | 7 [5, 10] | 0.124 |
| SAPS II | 42 [34, 52] | 42 [34, 52] | 42 [33, 51] | 0.128 |

Abbreviations: Aids: acquired immune deficiency syndrome, MAP: mean arterial pressure, SpO_2_: oxygen saturation, WBC: white blood cell, BUN: blood urea nitrogen, INR: international normalized ratio, PT: prothrombin time, PTT: partial thromboplastin time, RRT: renal replacement therapy, SOFA: sequential organ failure assessment, Scr: serum creatinine, SAPS II: Simplified Acute Physiology Score II.

**Table S3. Clinical features used for developing the models.**

| **Variables** |
| --- |
| **Demographic features** |
| Age |
| Weight |
|  |
| **Chronic disease history** |
| Cerebrovascular disease |
| Diabetes |
| Rheumatic disease |
| Paraplegia |
| Liver disease |
| Cancer |
|  |
| **Vital signs** |
| Heart rate |
| Respiratory rate |
| Body temperature |
|  |
| **Laboratory results** |
| Scr |
| Serum chloride |
| Hemoglobin |
| Platelets |
| Anion gap |
| WBC |
| INR |
| Serum sodium |
| BUN |
| PTT |
|  |
| **Urine output** |
|  |
| **Severity scores of illness** |
| SOFA |
| SAPS II |

Abbreviations: WBC: white blood cell, BUN: blood urea nitrogen, INR: international normalized ratio, PTT: partial thromboplastin time, Scr: serum creatinine, SOFA: sequential organ failure assessment, SAPS II: Simplified Acute Physiology Score II.


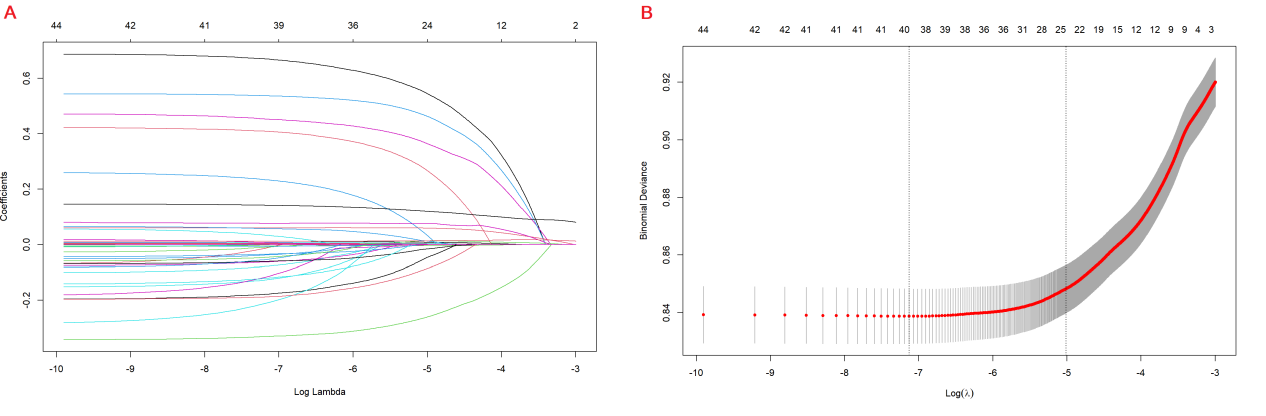


**Fig. S1. Feature selection using the LASSO regression model**

(A) LASSO coefficient profiles of the 44 baseline features.

(B) Tuning parameter (λ) selection in the LASSO model used 10-fold cross-validation via minimum criteria.

Abbreviations: LASSO: least absolute shrinkage and selection operator.

**
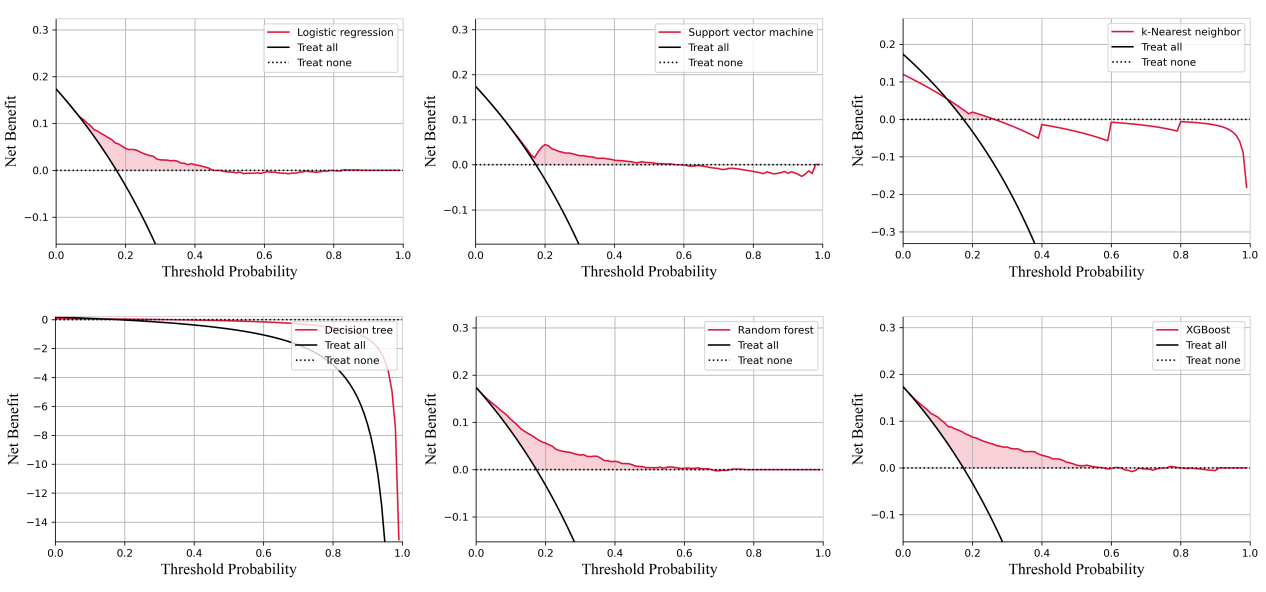
**

**Fig. S2 The decision curve analysis curves for the six models**

**
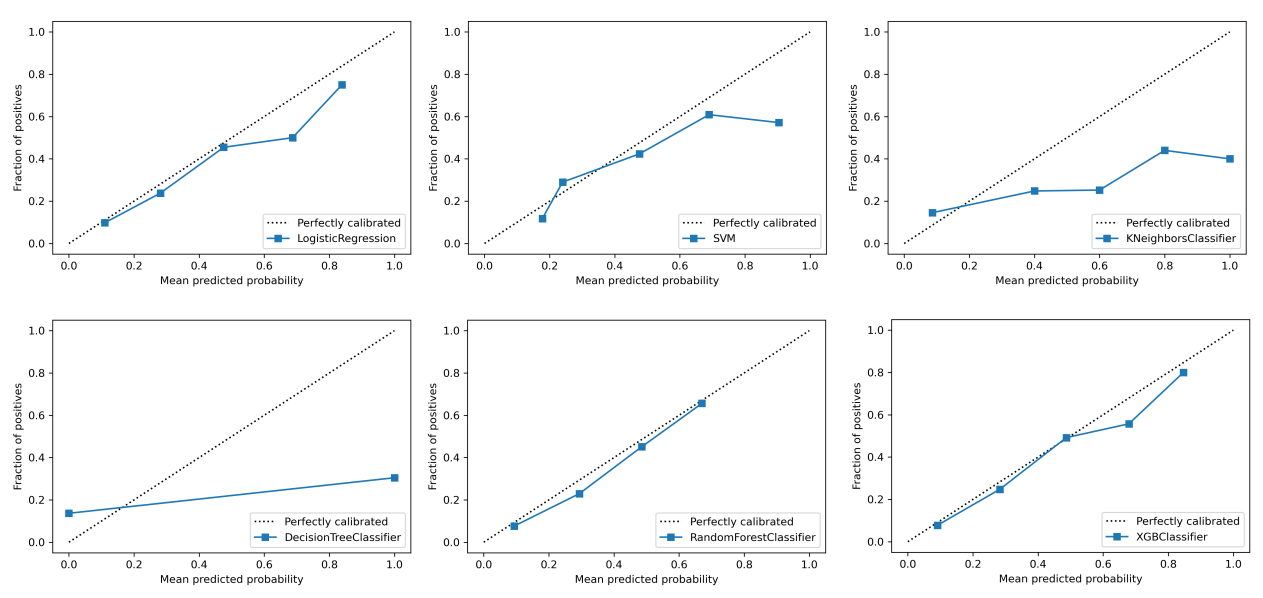
**

**Fig. S3 The calibration** **curves for the six models**

Abbreviations: SVM: support vector machine.

**
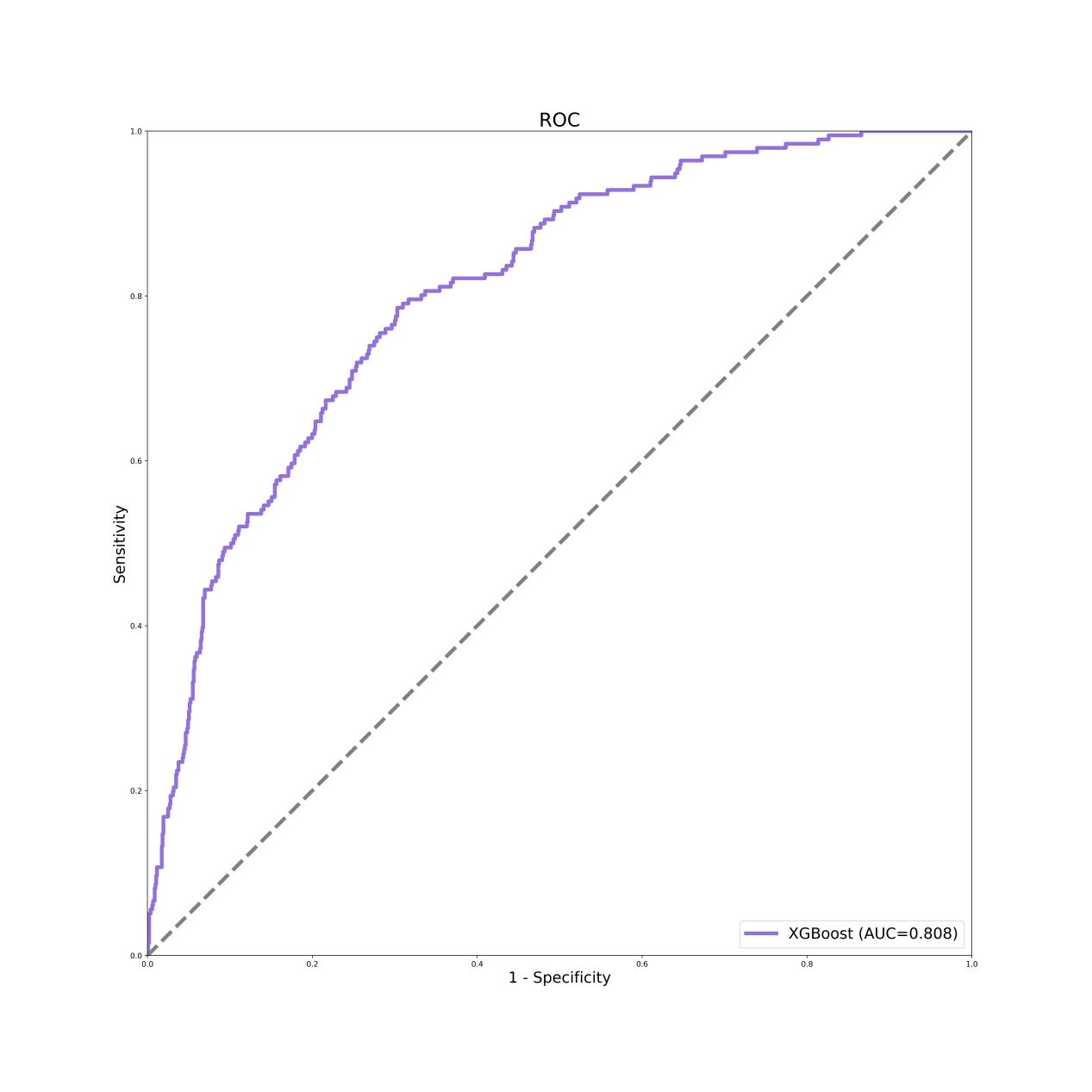
**

**Fig. S4 ROC curves of XGBoost models for predicting in-hospital mortality for patients without renal disease**
